# Whole-Brain Functional Networks for Phonological and Orthographic Processing in Chinese Good and Poor Readers

**DOI:** 10.3389/fpsyg.2019.02945

**Published:** 2020-01-14

**Authors:** Jing Yang, Li Hai Tan

**Affiliations:** ^1^Bilingual Cognition and Development Lab, Center for Linguistics and Applied Linguistics, Guangdong University of Foreign Studies, Guangzhou, China; ^2^Center for Brain Disorders and Cognitive Science, Shenzhen University, Shenzhen, China; ^3^Center for Language and Brain, Shenzhen Institute of Neuroscience, Shenzhen, China

**Keywords:** dyslexia, phonological deficit, orthographic deficit, Chinese, functional brain network

## Abstract

The neural basis of dyslexia in different languages remains unresolved, and it is unclear whether the phonological deficit as the core deficit of dyslexia is language-specific or universal. The current functional magnetic resonance imaging (fMRI) study using whole-brain data-driven network analyses investigated the neural mechanisms for phonological and orthographic processing in Chinese children with good and poor reading ability. Sixteen good readers and 16 poor readers were requested to make homophone judgments (phonological processing) and component judgments (visual-orthographic processing) of presented Chinese characters. Poor readers displayed worse performance than the good readers in phonological processing, but not in orthographic processing. Whole-brain activation analyses showed compensatory activations in the poor readers during phonological processing and automatic phonological production activation in the good readers during orthographic processing. Significant group differences in the topological properties of their brain networks were found only in orthographic processing. Analyses of nodal degree centrality and betweenness centrality revealed significant group differences in both phonological and orthographic processing. The present study supports the phonological core deficit hypothesis of reading difficulty in Chinese. It also suggests that Chinese good and poor readers might recruit different strategies and neural mechanisms for orthographic processing.

## Introduction

Developmental dyslexia, or in short, dyslexia, is characterized by a severe reading acquisition disorder that cannot be explained by general intelligence impairment, lack of education opportunities, or any sensory or neurological disorders (American Psychiatric Association, 2013). It is a widespread reading disorder that affects word recognition, decoding, and spelling abilities in 5–17% of the population, regardless of cultural or language backgrounds (Shaywitz et al., 1998; Ziegler et al., 2003; Siok et al., 2008; Cao et al., 2017). Phonological deficits, including impaired phonological representation and speech sound processing, are presented in the majority of dyslexics (Ziegler and Goswami, 2005) and therefore the phonological deficit hypothesis has been the most popular hypothesis about the cause of dyslexia (Rack et al., 1992; Pennington and Lefly, 2001; for a recent review, see Paulesu et al., 2014). This hypothesis posits that dyslexics are impaired in their phonological representation and their ability to process and manipulate speech sounds (e.g., Shankweiler and Lundquist, 1992; Ziegler and Goswami, 2005), which adversely affects the development of mapping between written forms (graphemes) and speech sound (phonemes) and hinders reading development (Snowling, 1981, 1998; Muter et al., 1998; Ramus and Szenkovits, 2008; Hulme et al., 2012; Castles and Friedmann, 2014).

### Phonological and Orthographic Deficits in Dyslexia

There is a tremendous amount of research on the brain mechanism of phonological processing deficits in dyslexics, and how such deficits affect reading development and might be relieved by phonological training (e.g., Shaywitz et al., 1998; Brunswick et al., 1999; Demb et al., 1999; Temple et al., 2001; Gaab et al., 2007; Cao et al., 2008; Tanaka et al., 2011; Steinbrink et al., 2012; Zhang et al., 2018). Most of the neuroimaging studies to date have investigated neural mechanism of dyslexia using visual word/pseudoword tasks and found reduced brain activation in the left temporo-parietal and temporo-occipital region in dyslexics speaking alphabetic languages (e.g., Rumsey et al., 1997; Paulesu et al., 2001, 2014; Schulz et al., 2009; van der Mark et al., 2009; Desroches et al., 2010; Pecini et al., 2011; Tanaka et al., 2011). The activation of the left inferior frontal gyrus (IFG) in dyslexics, however, increased in some studies (Shaywitz et al., 1998; Hoeft et al., 2007; MacSweeney et al., 2009) and decreased in other studies (Brambati et al., 2006; Cao et al., 2006; Booth et al., 2007; Wimmer and Schurz, 2010). Richlan et al. (2011) in a meta-analysis examined left temporo-parietal dysfunction for phonological deficits in dyslexic children and left ventral temporo-occipital dysfunction for visual-orthographic deficit in dyslexic adults. They found decreased activation of left ventral temporo-occipital region only in dyslexic adults.

Phonological deficits, however, are not the only problem in dyslexia. For example, Denckla and Rudel (1976) first reported picture naming problems in many people with dyslexia, who were slower than the normal when asked to rapidly name visual stimuli (for an overview, see Wolf et al., 2000). Wolf and Bowers, therefore, developed the double deficit hypothesis, which postulates that some people with dyslexia had a second independent naming speed deficit, which causes slower cross-modal matching of visual symbols and phonological codes, and therefore also causes reading problems (e.g., Bowers and Wolf, 1993; Wolf and Bowers, 1999; Vaessen et al., 2009).

Dyslexia is also suggested to be associated with orthographic deficits. First, rapid naming deficits seems to be quite universal among dyslexics in many languages, but phonological awareness deficit, difficulty to recognize and work with sounds in spoken language, are more common in opaque alphabetic languages (e.g., English) than transparent alphabetic languages (e.g., Italian) or non-alphabetic languages (e.g., Chinese) (e.g., Huang and Hanley, 1995; Ho et al., 2002; Ziegler et al., 2003; Tan et al., 2005; Vidyasagar and Pammer, 2010). Secondly, dyslexics exhibit deficits in processing letter strings in tasks with minimal phonological or lexical involvement, such as searching for a target letter in a string of consonants (e.g., Hawelka et al., 2006; Bosse et al., 2007; Collis et al., 2013). Ziegler et al. (2010) reported that dyslexics performed significantly worse than age-matched controls with letter and digit strings but not with symbol strings. The authors suggest that these deficits cannot be explained by weak reading experience in dyslexics, or dysfunctional visual attention processing, and reflect a deficit in processing a string of letters in parallel, probably due to difficulty in the coding of letter position. Finally, some neuroimaging studies have also found dyslexics show less activation than the normals in left fusiform gyrus, a system specialized for processing the orthographic structure of well-learned visual word forms (Rumsey et al., 1997; Brunswick et al., 1999; Temple et al., 2001; Shaywitz and Shaywitz, 2003; Cao et al., 2006; van der Mark et al., 2009; Boros et al., 2016). For example, Desroches et al. (2010) reported reduced brain activation in the fusiform gyrus in dyslexics compared with the normals during an auditory rhyming task. The brain activation in left fusiform gyrus of the dyslexics correlated significantly and positively with their non-word reading performance. The authors, therefore, suggest that dyslexics were impaired in the access to orthography and the integration of orthographic and phonological processing.

The dysfunction activation of fusiform gyrus may be secondary to a primary dysfunction of the temporo-parietal region (Boros et al., 2016). Orthographic deficits in dyslexics increase the difficulty of selecting graphemes in fusiform gyrus, which are the input to the grapheme-phoneme processing and phonological decoding system in the temporo-parietal region. Therefore dyslexia might be characterized by the co-existence of orthographic and phonological processing difficulties (Siok et al., 2009).

### Dyslexia in Chinese

Siok et al. (2004) found that Chinese dyslexic children reading in Chinese did not show underactivation in the left temporo-parietal regions as typically shown in studies of alphabetic languages. They reported reduced activity at Brodmann’ area (BA) 9 in the left middle frontal gyrus (MFG), an area involved in syllabic processing of phonology (Siok et al., 2003). This study provides the first neural evidence to support previous findings of phonological awareness predicting reading development of Chinese children (e.g., McBride-Chang et al., 2008; Pan et al., 2011, 2015) and impaired phonological awareness in Chinese dyslexic children (e.g., Ho and Lai, 1999; Ziegler and Goswami, 2005), but also challenges the biological unity of dyslexia.

Unlike alphabetic languages, Chinese is a logographic language, in which the basic orthographic units, the characters, map onto morphemic meanings and to monosyllables with Chinese four tones in the spoken language. Therefore, Chinese reading needs a fine-grained visuospatial analysis to access characters’ phonology and meaning. Chinese readers must learn the character phonology at the syllabic level as a whole by rote, and they might need additional strategies like writing to learn those characters (Siok et al., 2004; Tan et al., 2005; Ziegler, 2006; Cao et al., 2013; cf. Bi et al., 2009).

Siok et al. (2009) compared Chinese dyslexic children and normal children in a decision task of Chinese character physical size. The normal showed greater activation than the dyslexic in the right inferior parietal lobe; the dyslexics, however, had more neural response than the normal participants in left inferior parietal lobe and lingual gyrus subserving visual analysis. According to the authors, phonological and orthographic disorders co-exist in the majority (83.33%) of Chinese dyslexics. The findings of Siok et al. (2009) are congruent with earlier behavioral reports of visual-orthographic deficits in Chinese dyslexics (Huang and Hanley, 1995; Ho et al., 2002).

Hu et al. (2010) examined brain activations of Chinese dyslexics, English dyslexics, English normal readers, and Chinese normal readers in a semantic decision task on written words. They found Chinese and English dyslexic adolescents had common underactivation than their normal controls in the left angular gyrus, left middle frontal, posterior temporal, and occipito-temporal regions. The authors suggest commonalities of manifestation of dyslexia in Chinese and English population, which could be influenced by readers’ cognitive ability and learning environment, as is congruent with Ziegler’s claim on the universal phonological core deficit of dyslexia (Ziegler, 2006).

### Brain Connectivity in Dyslexia

A significant trend in cognitive neuroscience today is the brain connectivity approach, which explores the functional or structural connectivity patterns of brain regions that support cognitive or linguistic processing. A few studies have adopted this approach toward dyslexia.

In their pioneer work on dyslexia and connectivity, Horwitz et al. (1998), using positron emission tomography (PET) found that the dyslexics’ left angular gyrus is functionally disconnected from the extrastriate occipital and temporal lobe regions during single-word reading, compared with the normal adults. They suggest a disconnected brain network in dyslexia. More recently, Boets et al. (2013) examined whether dyslexics’ phonological deficits are caused by impaired phonological representation or by dysfunctional retrieval of phonological representations. They found that adult dyslexics have intact phonetic representations. Their functional and structural connectivity between the bilateral auditory cortices and the left IFG, however, is significantly smaller than the normal adults. Cao et al. (2017) focused on the phonological deficits of Chinese dyslexic children, who were asked to perform an auditory rhyming judgment task. They found that Chinese dyslexics were impaired in the left dorsal IFG and they had more reliance on the right precentral gyrus than the normal controls as a compensatory strategy. Their functional connectivity analyses showed that connectivity between the left STG and the left dorsal IFG was sensitive to task performance and/or reading skill rather than being dyslexic or not. In a functional connectivity study of orthographic processing of dyslexia, van der Mark et al. (2009) focused on the role of the left visual word form area in temporo-occipital area and found a significant disruption of the functional connectivity between the visual word face area (VWFA) and left inferior frontal and left inferior parietal language areas in the dyslexic children. They suggest that dyslexia is associated with impaired automatic visual word processing, along with deficits in orthographic and phonological processing. The studies mentioned above were based on the analysis of regions of interests (ROIs), and therefore their results depend on the selected regions, which are arbitrary decisions by the authors. Finn et al. (2014) adopted a whole-brain, data-driven analysis to examine the functional networks in dyslexics. They found reduced connectivity in the visual word-form areas and increased right-hemisphere connectivity in the dyslexics compared with the normal adults. However, the parcellations in both the younger reader and older reader groups were generated from their groups of normal participants with limited group size (30–45). Their data analysis focused on group differences in regional connectivity and did not compare the topological features of brain networks.

### The Present Study

The present functional magnetic resonance imaging (fMRI) study investigated phonological processing and orthographic processing in Chinese children with good and poor reading ability to improve the current understanding of the universal neural mechanism for dyslexia. All participants were asked to perform a homophone judgment task (phonological processing) and a component search task (visual-orthographic processing) inside the fMRI scanner. We examined group differences in their whole-brain activation and analyzed the topological features of their functional brain networks to reveal the neural mechanisms for phonological and visual-orthographic processing in Chinese good and poor readers.

## Materials and Methods

### Participants

Five hundred and twenty-four 4th and 5th graders from the Beijing Yongtai Primary School in China participated in the screening for good readers and poor readers. Since there was no standardized dyslexia screening assessment or Chinese reading ability test in mainland China, we measured the children’s reading ability using a character-reading test, their Chinese teachers’ evaluation, and their school performance in the Chinese language course. This character-reading test was adapted from the reading test to evaluate Chinese children’s reading ability by Tan et al. (2005), comprised 120 Chinese characters from the textbooks for third to fifth graders (40 characters for each grade) and 40 characters beyond the primary school textbooks. The 160 characters were printed on a standard A4 sheet, listed in 16 rows and 10 columns, and arranged from easy to difficult based on grade level. Each participant was asked to read out the 160 characters as accurately and as fast as possible with a time limit of 90 s. Their name accuracy (number of characters correctly named) represented their reading performance: Poor readers had reading scores 1.5 standard deviations below the mean; good readers had reading scores 1.5 standard deviations above the mean. Their reading performance was congruent with the evaluation from their Chinese teachers and their school performance in the Chinese course. Seventeen children with dyslexia and 16 controls participated in the present fMRI study. One participant from the normal group was excluded because of neurological disease found during the fMRI scans.

The reading performance (Mean ± SD = 115.75 ± 13.57) of the 16 participants in the normal group (9 men, average age = 10 years 1 month) was significantly better than that (Mean ± SD = 35.63 ± 13.59) of the 16 participants in the dyslexic group (12 men, average age = 10 years 6 months), *t*_30_ = 16.69, *p* < 0.001. All participants, who were native speakers of Chinese and right-handed (Oldfield, 1971), had average and matched non-verbal intelligence according to their performance in the Raven’s Progressive Matrices (Raven et al., 1998) (good readers, Mean ± SD = 68.44 ± 15.78; poor readers, Mean ± SD = 75 ± 16.73; *t*_30_ = −1.141, *p* = 0.26). This fMRI study was approved by the Beijing Institutional Review Board at the Chinese Academy of Sciences. Written informed consent was obtained from each child and his/her legal guardians, mostly their parents.

### Stimuli and Procedure

In this blocked-design fMRI study, both groups underwent a phonological session and a visual-orthographic session. During the phonological session, participants performed a homophone judgment task in experimental blocks: they were asked to judge whether the characters (e.g., “
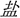
” sounds/*yan2*/and means “*salt*”) presented had the same pronunciation including tones with the “pinyin^1^” (e.g., “*yán*” sounds/*yan2*/) specified at the instruction page before each experimental block. During the visual-orthographic session, participants completed a component judgment task: they were asked to identify whether the characters (e.g., “
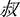
” sounds/*shu1*/and means “*uncle*”) presented contained a radical (e.g., “
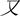
”) specified at the instruction page before each experimental block. Chinese orthographic processing involves visuospatial analysis of Chinese characters and the application of orthographic rules (orthographic awareness). Component search task (orthographic search) asks participants to judge whether a character contained a designated a radical component and has been used as Chinese visual-orthographic processing task in previous studies (e.g., Siok and Fletcher, 2001; Ding et al., 2003).

Both sessions included four experimental blocks (homophone judgment/component search): each block began with a 2-s instruction and included eight trials; each trial started with a 500-ms presentation of Chinese character at the center of the screen, followed by a 2500-ms blank screen for responses. All the experimental blocks were interleaved with 12-s fixation blocks. Participants made “Yes” or “No” responses by clicking right or left buttons with their index fingers on a control box compatible with the fMRI scanner. The Chinese character stimuli, selected from the children’s textbooks, were matched between experimental tasks in terms of character frequency and visual complexity (strokes).

### MRI Acquisition

MRI images were acquired on a Siemens Vision Magnetom 3.0-tesla scanner with a circularly polarized head coil at the Beijing MRI Imaging Center. Before the fMRI scans, all participants underwent a practice session and were visually familiarized with all the procedures and experimental conditions. They lay supine in the scanner with plastic ear-canal molds and looked up through a prism at a screen at the end of the scanner, while their heads were immobilized by a tightly fitting, vacuum pillow. A T_2_^∗^-weighted gradient-echo planar imaging (EPI) sequence was used for fMRI scans: slice thickness = 4 mm, in-plane resolution = 3.125 × 3.125 mm^2^, and TR/TE/flip angle = 2000 ms/30 ms/90°. The field of view (FOV) was 200 × 200 mm^2^, and the acquisition matrix was 64 × 64. Thirty-two contiguous axial slices were acquired parallel to the anterior commissure–posterior commissure (AC–PC) line covering the whole brain.

### Data Analyses

#### Whole-Brain Activations

SPM 12 was used for image preprocessing and statistical analyses^2^. Functional images from each participant were realigned and normalized to an EPI template based on the ICBM152 stereotactic space, an approximation of canonical space (Talairach and Tournoux, 1988). The images were further re-sampled into 3 mm × 3 mm × 3 mm cubic voxels and spatially smoothed with an isotropic Gaussian kernel (6 mm full width at half-maximum). After motion-correction, the first three images (dummy images), corresponding to the period of transient hemodynamic change that occurred before the experimental trials, were discarded. The general linear model included 12 motion regressors was used to estimate the condition effect of each individual, while boxcar convolved with the canonical hemodynamic response function was selected as a reference function. Adjusted mean images were created for each condition after removing global signal and low-frequency covariates, using a high-pass filter with a cut-off of 128 s. Contrast images of homophone judgment minus fixation in phonological scanning session and component judgment minus fixation in visual-orthographic session were computed, using a Student’s group *t-*test, which generated the statistical parametric maps of *t*-values. For each session, all the contrast estimates from dyslexic and normal groups were entered into a standard SPM second-level analysis with subjects treated as a random effect, using two-samples *T*-test to examine possible group differences in brain activations.

All the brain activations reported below were in MNI coordinate space and survived a corrected cluster-level threshold of *p* < 0.05 (single voxel *p* = 0.005, 10000 simulations, and a minimum cluster size of 25 voxels) using AlphaSim program in REST software (Song et al., 2011).

#### Network Construction

Functional brain networks for good readers and poor readers were constructed at the macroscale in which nodes represent brain regions, and edges present the statistical relationships of blood oxygenation level-dependent (BOLD) signals across different regions. Here, we used the 90 regions (45 for each hemisphere) of the atlas of Automated Anatomical Labeling (AAL) (Tzourio-Mazoyer et al., 2002) as nodes of the brain network. The averaged time series of all the voxels within each ROI was extracted in each individual. Edges, or interregional functional connectivity, were calculated using Pearson correlations between these regional task-related time series of all possible pairs of the 90 regions for each participant. The correlation coefficients were then transformed to *z*-scores via Fisher’s transformation to improve normality (Lowe et al., 1998). Thus each participant has a 90 × 90 correlation matrix for phonological and visual-orthographic sessions, respectively.

#### Network Analysis

##### Threshold selection

We constructed binary undirected functional networks using a sparsity threshold (5% ≤ sparsity ≤ 50%, interval = 5%) to comprehensively estimate topological properties covering a wide range of sparsity and remove spurious edges as much as possible (Yang et al., 2017; Zhu et al., 2017). Because the physiological interpretation of negative correlations is ambiguous (e.g., Murphy and Fox, 2017), functional connections with negative correlation values were not considered in the present analysis.

##### Network metrics

Our network analyses were performed in the GRETNA toolbox (Wang et al., 2015). We calculated both the global and node network metrics at each sparsity. These metrics included: (1) The “small-world” parameters of clustering coefficient (Cp), shortest path length (Lp), normalized clustering coefficient (γ), normalized shortest path length (λ), and small-worldness (σ); (2) Network efficiency measures of the local efficiency of the whole network (*E*_loc_) and the global efficiency of the network (*E*_glob_); (3) Nodal centrality degree and betweenness degree that reflect functional segregation and integration (Rubinov and Sporns, 2010).

##### Group comparisons based on topological metrics

To examine group differences of all the network metrics mentioned in the above section, two-sample *t*-test analyses were used for between-subject comparisons. To correct for multiple comparisons, we used a Bonferroni corrected threshold at the significance level of 0.05. The network results were visualized using BrainNet Viewer (Xia et al., 2013).

## Results

### Behavioral Results

**FIGURE 1:**
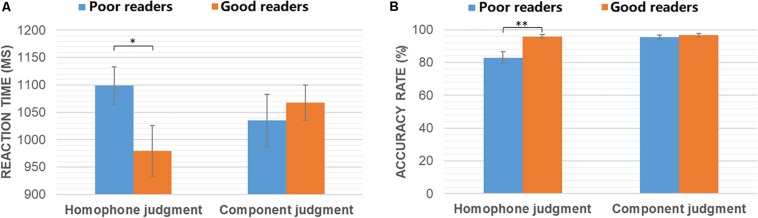
Reaction time **(A)** and accuracy rates **(B)** of poor readers and good readers in homophone judgment and component judgment task. ^∗^*p* < 0.05; ^∗∗^*p* < 0.005.

Independent-samples *T*-tests were conducted to compare the behavioral performance of good and poor readers in homophone judgment and component judgment tasks, respectively. As shown in Figure 1, poor readers were significantly slower (*t*_30_ = −2.08, *p* = 0.046) and less accurate (*t*_30_ = 3.31, *p* = 0.004) than the normals in the homophone judgment task. However, the two groups had similar performance in the component judgment task (ps > 0.05).

### Whole-Brain Activations

**FIGURE 2:**
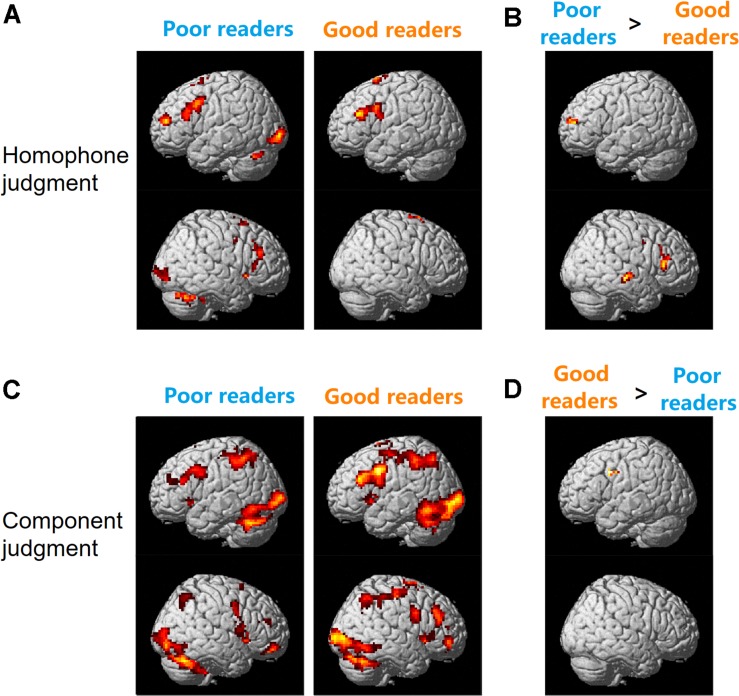
Brain activation for homophone judgment **(A,B)** and component judgment **(C,D)** task in Chinese poor readers and good readers. Poor readers showed more neural responses than the good readers in homophone judgment task **(B)** and the good readers involved more brain activation than the poor readers in component judgment task **(D)**.

As shown in Figure 2A, during the homophone judgment task, good readers recruited left MFG (BAs 9, 46), left IFG (pars triangularis, BA 45), and bilateral SMA (BAs 6, 8). In contrast, poor readers involved an extensive and symmetrical brain network, including the bilateral prefrontal cortex, insula, cingulate cortex, caudate nuclei, occipital regions, and cerebellum. Group comparisons showed poor readers had significantly more neural responses in the left anterior MFG, right IFG, right superior and middle temporal gyrus (MTG; Figure 2B). The good readers didn’t show more neural responses compared with the poor readers.

During the component judgment task, Chinese good readers showed brain activations in bilateral middle and inferior frontal gyri, precentral gyri, SMA, insula, cingulate cortex, basal ganglia, and thalamus. Bilateral superior and inferior parietal lobules, posterior temporal-occipital cortex, and cerebellum were also involved in this group. The poor readers showed neural responses in those regions similar to that of the good readers (Figure 2C). During the component judgment task (in contrast to the fixation baseline condition), the good readers had significantly more neural activity in the left premotor cortex (BA 6) than the poor readers (Figure 2D). All reported group differences in brain activation were summarized in Table 1.

**TABLE 1:** Significant differences between Chinese poor readers and good readers in brain activations for homophone judgment and component judgment task.

**Regions**	**L/R**	**BA**	**MNI**			***Z*-value**	**L/R**	**BA**	**MNI**			***Z*-value**
			***x***	***y***	***z***				***x***	***y***	***z***	
**Homophone judgment**			Good readers > Poor readers						Poor readers > Good readers			
Middle frontal gyrus		None					L	10	−30	57	15	3.59
Inferior frontal gyrus							R	45	48	27	9	4.18
Superior temporal sulcus							R	22	66	−15	−3	3.5
**Component judgment**												
Premotor cortex	L	6	−48	6	36	3.19	None					

*MNI, MNI coordinates. L, left hemisphere; R, right hemisphere.*

### Network Metrics

**FIGURE 3:**
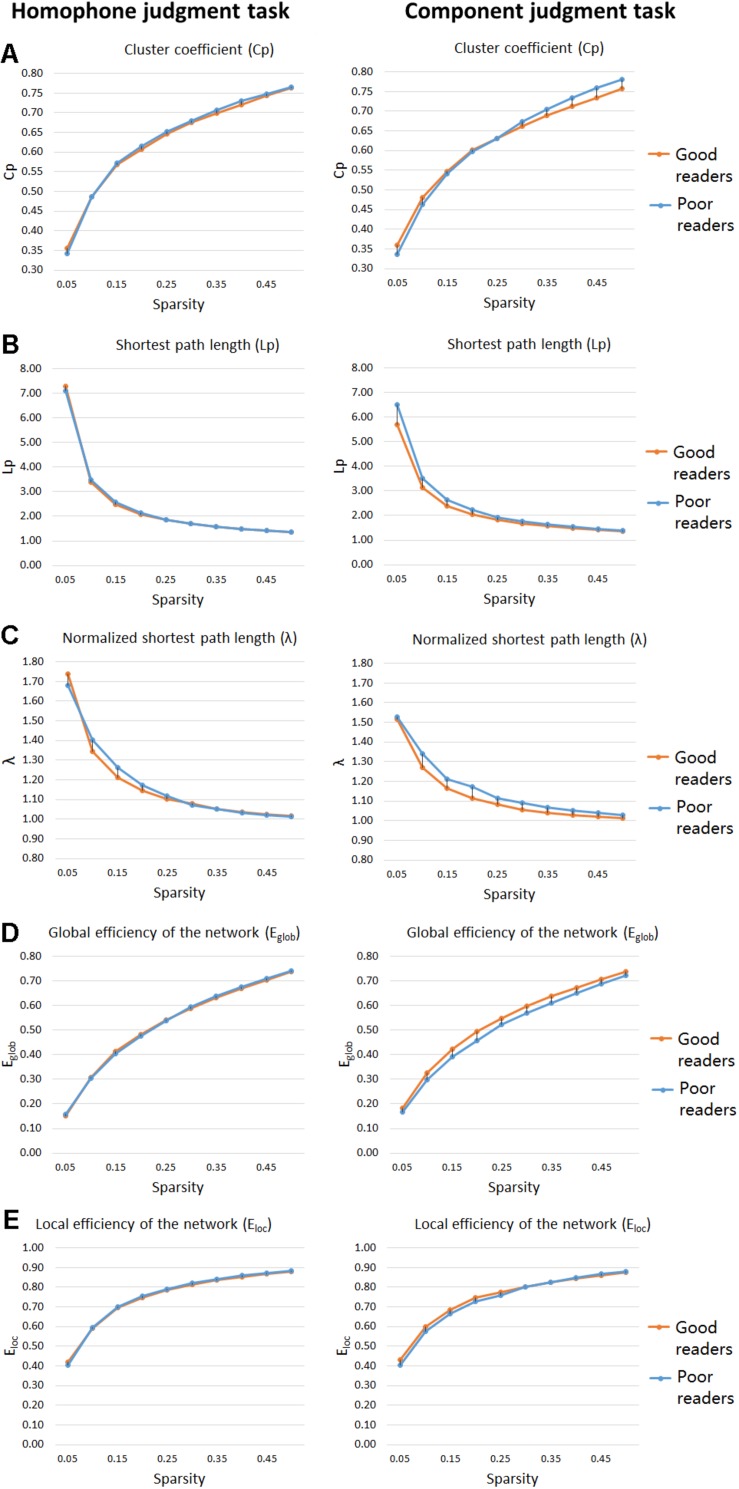
“Small-world” parameters and network proficiency metrics in the defined threshold range (0.05–0.5). Two-sample *t*-tests show that poor readers are different from the good readers in Cp **(A)**, Lp **(B)**, λ **(C)**, *E*_glob_ **(D)**, and *E*_loc_ **(E)** metrics of functional networks for orthographic processing (component judgment task), but not those for phonological processing (homophone judgment task). There are no group differences in *E*_loc_, for both phonological and orthographic processing. Cp, network clustering coefficient; Lp, shortest path length; λ, normalized shortest path length, *E*_glob_, global efficiency of the network; *E*_loc_, local efficiency of the network.

As shown in Figures 3A–C, significant group differences were found between their clustering coefficient (Cp), shortest path length (Lp), and normalized shortest path length (λ) of functional networks for visual-orthographic processing (component judgment task), but not for phonological processing (homophone judgment task). To be specific, during visual-orthographic processing, the brain networks of the dyslexic children displayed significantly higher Cp at the sparsity threshold of 45% (dyslexics, 0.76 ± 0.04; normal, 0.73 ± 0.02; *t* = −2.14, *p* = 0.04). They also had higher values of Lp than the normals for thresholds between 25 and 45%; the groups were significantly different in their λ at the thresholds of 30, 35, 40, and 45% (ps < 0.05).

### Network Efficiency

For the homophone judgment task, there were no significant group differences in their local efficiency (*E*_loc_) or global efficiency (*E*_glob_). For the component judgment task, the good readers displayed higher global efficiency than the poor readers at the thresholds between 20 and 50% (ps < 0.05). No group difference was found for local efficiency in the component judgment task.

### Nodal Centrality Degree

**FIGURE 4:**
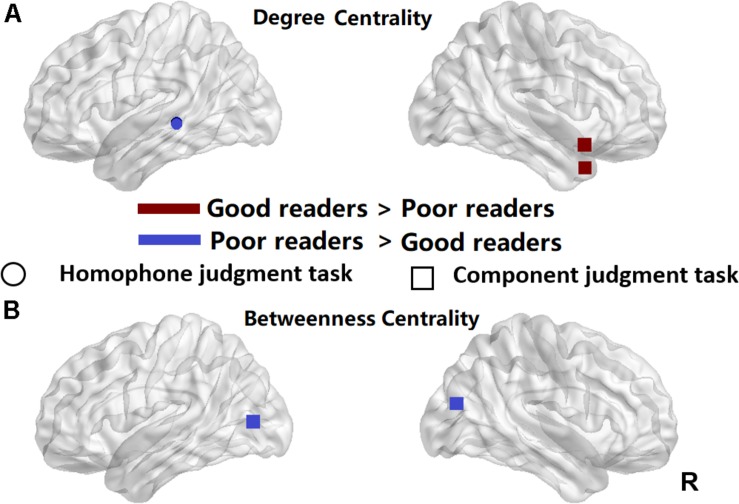
Significant differences between the poor readers and the good readers in their nodal degree centrality **(A)** and betweenness centrality at the sparsity threshold of 0.05 **(B)**. Red, the good readers children had higher nodal centrality than the poor readers; Blue, the poor readers had higher nodal centrality than the good readers. R, right hemisphere. Circle, homophone judgment task; Square, component judgment task.

We used two-sample *t*-tests to examine group differences in nodal centrality measures of degree centrality and betweenness centrality at the strongest threshold (sparsity = 5%) so that all/most of the nodes were connected (Table 2). The poor readers displayed higher degree centrality in left middle temporal gyrus (MTG) during homophone judgment task compared with the good readers, who displayed higher degree centrality than the former in the right temporal pole (TP; superior and middle temporal gyri) during component judgment task (Figure 4A). As shown in Figure 4B, poor readers showed significantly higher betweenness centrality than the good readers in left calcarine fissure and right middle occipital gyrus in component judgment task. There were no significant group differences in betweenness centrality in homophone judgment task.

**TABLE 2:** Significant differences between Chinese poor readers (Poor) and good readers (Good) in nodal degree centrality and betweenness centrality in homophone judgment task (Homophone) and component judgment task (Component) at the sparsity threshold of 5%. MNI, MNI coordinates.

**Centrality measures**	**Node**	**Volume-based ROI (MNI)**			**Voxel size**	***T***	***P***
		***x* (mm)**	***y* (mm)**	***z* (mm)**			
**Degree centrality**							
Homophone: Poor > Good	Middle temporal gyrus	−56	−34	−2	1439	–2.28	0.03
Component: Good > Poor	Temporal pole: superior temporal gyrus	48	15	−17	400	2.33	0.026
	Temporal pole: middle temporal gyrus	44	15	−32	349	2.05	0.049
**Betweenness centrality**							
Component: Poor > Good	Calcarine fissure	−7	−79	6	648	–2.26	0.03
	Middle occipital gyrus	37	−80	19	595	–2.25	0.03

**T*, two-sample *t*-test; *P*, all *p*-values less than 0.05.*

## Discussion

The present fMRI study using a whole-brain data-driven network approach examined the neural correlates of phonological and visual-orthographic processing in Chinese good readers and poor readers, who were forth or fifth graders matched in age and non-verbal intelligence. Our behavioral data showed that poor readers made more errors and responded more slowly than the good readers in phonological processing (homophone judgment task). There were no group differences in orthographic processing (component judgment task) at the behavioral level. Our behavioral findings are consistent with the phonological deficit hypothesis of dyslexia and suggest no orthographic deficits in Chinese children with reading difficulties (poor readers). Whole-brain activation analyses, however, revealed the poor readers compared with the good readers had hyperactivity in left MFG (BA 10), right IFG (BA 45), and right superior temporal sulcus (STS) (BA 22) during phonological processing, and hypoactivity in the left premotor cortex (BA 6) during visual-orthographic processing. In line with poor readers’ behavioral deficits in phonological processing, the aberrant brain activations for phonological processing in Chinese poor readers suggests neurological disorder underlying the phonological processing of dyslexics. For visual-orthographic processing, the two groups might both function normally with different neural correlates. To provide a complete picture of the brain connectivity profiles of Chinese children with reading difficulties, we examined the topological features of their functional brain networks. During phonological processing, there were no significant group differences in measures of functional segregation (cluster coefficient, Cp) or functional integration (shortest path length, Lp, or the normalized shortest path length, λ). Nor were they different in their values of the global efficiency (*E*_glob_) or local efficiency (*E*_loc_). In visual-orthographic processing, poor readers displayed larger functional segregation (Cp) and less functional integration (Lp, λ) than good readers, who showed higher global efficiency (*E*_glob_).

Further analyses of node centrality showed that during phonological processing, poor readers had a larger value of degree centrality at the left posterior MTG (pMTG) than the good readers, implying its more interactive role as a hub in network of dyslexics. During visual-orthographic processing, the good readers showed more centrality degree in the right TP and less betweenness centrality in the left calcarine fissure and middle occipital gyrus. Based on previous findings and our data reported above, we suggest a phonological core deficit of Chinese dyslexia and different visual-orthographic processing mechanisms in Chinese good and poor readers.

### Impaired Phonological Processing, More Efforts on Cognitive Control and Semantic Processing for an Intact Functional Brain Network

Consistent with previous reports on the phonological deficits of Chinese dyslexia, the present study showed that Chinese children with reading difficulties performed worse than the good readers in the homophone judgment task. We didn’t find underactivation in either the left MFG, IFG, temporo-parietal region or fusiform gyrus in the poor readers as reported in previous studies of dyslexia, in particular, Chinese dyslexia (e.g., Shaywitz et al., 1998; Paulesu et al., 2001; Temple et al., 2001; Cao et al., 2008, 2017; Tanaka et al., 2011). Instead, hyperactivation was found in the left anterior prefrontal cortex (aPFC), right IFG, and right posterior STS (pSTS) in poor readers. The aPFC is responsible for integrating outcomes of separate cognitive operations in the pursuit of a higher behavioral goal (for a review, see Ramnani and Owen, 2004) and the right IFG is involved in cognitive control and is recruited when important cues are detected (e.g., Hampshire et al., 2010). Therefore, the larger involvement of left aPFC and right IFG might indicate Chinese poor readers recruited more cognitive control and outcome integration as a compensatory strategy, which is domain-general. Studies on dyslexia have reported reduced gray matter volume in dyslexic readers in the right STG and left STS (e.g., Richlan et al., 2013) and symmetrically distributed gray matter in STS (Dole et al., 2013). The underactivation of left temporo-parietal region is also well-documented in studies of dyslexia, especially in alphabetic languages (Rumsey et al., 1997; Paulesu et al., 2001, 2014; Schulz et al., 2009). We hypothesize that in addition to cognitive control and feedback strategies, our poor readers might have recruited the right homologous site of left pSTS for semantic association and memory, as the left pSTS is a cortical hub for semantic processing and the extraction of meaning from multiple sources of information (Liebenthal et al., 2014).

Although there were no significant group differences in the small-world properties (Cp, Lp, λ, *E*_glob_, and *E*_loc_) of their functional networks for phonological processing, the poor readers had a larger value of degree centrality than the good readers in the left pMTG, which contributes to controlled retrieval of conceptual knowledge (Davey et al., 2016). With this compensatory strategy, poor readers had similar global and local brain network efficiency, despite their poor performance in the phonological task.

### Functioning Orthographic Processing, Less Automatic Phonological Retrieval and Multimodal Integration, More Delays in Visual Analysis Hub, and Low Efficient Brain Network

Our studies didn’t find behavioral deficits of Chinese poor readers in visual-orthographic processing. However, they engaged different brain activation and functional network to complete the same task as good readers did. Specifically, when the poor readers were fully occupied by the visual-orthographic processing task, the good readers automatically and efficiently activated the phonology of the presented character stimulus, and displayed more brain activation in the left premotor cortex, which is involved in speech production, especially articulation (e.g., Small et al., 1998; Donnan et al., 1999).

It is possible that Chinese poor readers recruited different neural mechanisms for visual-orthographic processing because they tend to have larger values of cluster coefficient and shortest path length, which also brings them a disadvantage in global efficiency compared with the good readers. The global efficiency of a network is a measure of network integration (Achard and Bullmore, 2007; Rubinov and Sporns, 2010), implying poor readers have a lower integration of functional network for visual-orthographic processing. The degree centrality analysis showed within the functional brain network of poor readers, the centrality of the right TP is less than that of the good readers. As bilateral TP are the core neural substrate for the formation of semantic representation (e.g., Lambon Ralph et al., 2009), our studies seem to suggest that the semantic representation in poor readers are not informative or complete as in the good readers. Meanwhile, betweenness centrality analysis found bilateral posterior visual cortex (calcarine fissure and middle occipital gyrus) play a more active role in information transportation of poor readers than in that of good readers during visual-orthographic processing, which indeed suggests more dependence on the visual neural correlates when poor readers perform the same visual-orthographic task as the good readers.

Our findings of the abnormal functional network for orthographic processing in Chinese children with reading difficulties are consistent with previous findings on the topological organization of brain structural network in Chinese dyslexic children (Liu et al., 2015). The authors using a similar whole-brain network analysis approach examined the structural brain network of Chinese dyslexics and found higher local specialization, a tendency of lower *E*_glob_ and prolonged characteristic path length in the dyslexic than the normal, supporting our findings of the functional networks in Chinese children with reading difficulties.

### Dynamic Brain Networks in Developmental Dyslexia

Using a whole-brain approach, the current study explored the differences between Chinese good and poor readers. Compared with previous studies in alphabetic languages, this study supports the phonological core deficit hypothesis of dyslexia and pointed out that behaviorally and neurologically dyslexics had manifestations of phonological processing deficits. Meanwhile, our results also imply distinct orthographic processing between Chinese good and poor readers, especially the inefficient functional brain network in poor readers during visual-orthographic processing.

The question remains: why abnormal brain activation and the inefficient brain functional network didn’t cause orthographic processing deficits in Chinese dyslexics, as they do with phonological processing. We hypothesize that the neural mechanism for reading including the functional brain network is dynamic and developing, and behavioral performance of poor readers can be improved.

Training studies on dyslexia have provided numerous evidence on the effects of therapy or remediation on dyslexia. For example, the Tallal–Merzenich team provided intensive auditory training in dyslexic children and showed how the training rewired the children’s brain (Merzenich et al., 1996; Tallal et al., 1996). Shaywitz et al. (2004) recruited second and third graders and administered phonologically mediated reading intervention to those with reading disabilities. Children who received the experimental intervention not only improved their reading performance but also showed increased brain activation in bilateral IFG, left STS, and temporo-occipital regions. Interestingly, Krafnick et al. (2011) reported gray matter volume changes in the left anterior fusiform gyrus/hippocampus, left precuneus, right hippocampus, and right anterior cerebellum during the intervention period. Those areas did not change after the training was stopped.

As we know, learning to read is associated with changes in brain activity. For example, Turkeltaub et al. (2003) in a cross-sectional fMRI study on subjects whose ages ranged from 6 to 22 years found reading acquisition is associated with increased activity in left MTG and IFG and decreased activity in the right inferior temporal regions. Learning to read also changes brain connectivity in dyslexics. Morken et al. (2017) traced reading process of dyslexics during their reading development. In this longitudinal study, participants were scanned through Pre literacy (6 years old), Emergent Literacy (8 years old), and Literacy (12 years old) stages. This study is the first fMRI study tracing the effectivity connectivity in dyslexics. Using Dynamic Causal Modelling (DCM) approach, they found different effectivity patterns in readers with and without dyslexia at age 6 and 8, but 12, implying by age 12, dyslexics reached functional, albeit poor reading skill with normalized effectivity close to the normal.

In the current study, participants were fourth and fifth graders, who had at least 5 years of experience in Chinese character writing and their Chinese literacy is close to the Literacy stage in Morken et al. (2017). It is possible that poor readers have orthographic deficits in their early years of Chinese reading acquisition. After they begin to receive school education, they are asked to do a lot of practice on Chinese writing and spelling to memorize Chinese words by rote in school and after school. Not surprisingly, Chinese writing can predict children’s reading development (e.g., Tan et al., 2005; Cao et al., 2013). With reading development and intensive writing practice, their visual-orthographic processing, which was at a disadvantage in the beginning, might be improved to the extent that the differences between good and poor readers are not significant in terms of their behavioral performance. Only by neuroimaging techniques, we were able to reveal group differences in their neural substrates for visual-orthographic processing.

Meanwhile, the phonological deficit as the core deficit of dyslexia is not alleviated as reading skill approve. Their behavioral performance in phonological manipulation is still significantly different from good readers. Most of the intervention studies on dyslexia adopt the phonological-based training program. If more phonological-based training is used in the classroom setting, phonological deficits might be less in dyslexics as their reading literacy increases.

## Conclusion

This study used a whole-brain data-driven network approach to examine the topological features of functional brain networks for phonological and visual-orthographic processing in Chinese good and poor readers. Our results suggest phonological deficits and aberrant neural mechanisms in Chinese poor readers, implying a language-universal phonological deficit in dyslexia. Our findings also indicate good and poor readers rely on different neural mechanisms or strategies in visual-orthographic processing to arrive at similar behavioral performance. To fully understand how phonological processing and visual-orthographic processing progress as reading literacy develops, we will need longitudinal studies tracking the reading development of dyslexics in typical classroom settings using brain imaging techniques.

## Data Availability Statement

The datasets generated for this study are available on request to the corresponding author.

## Ethics Statement

The studies involving human participants were reviewed and approved by the Key Laboratory of Brain and Cognitive Sciences at The University of Hong Kong. Written informed consent to participate in this study was provided by the participants’ legal guardian/next of kin.

## Author Contributions

LT and JY conceived the presented study, collected the data, discussed the results, and contributed to the final manuscript. JY performed the data analyses.

## Conflict of Interest

The authors declare that the research was conducted in the absence of any commercial or financial relationships that could be construed as a potential conflict of interest.

## Footnotes

1 “Pinyin” is an alphabetic phonetic system mainly used in Mainland China to represent pronunciation of Chinese characters in Putonghua, standard spoken language in Mainland China. All children (6–7 years old) enrolled in primary school education at Mainland China are trained in Pinyin for 6–8 weeks before starting to learn Chinese characters.

2 http://www.fil.ion.ucl.ac.uk/spm
